# Evaluation of the Effect of Different Growth Media and Temperature on the Suitability of Biofilm Formation by *Enterobacter cloacae* Strains Isolated from Food Samples in South Africa

**DOI:** 10.3390/molecules18089582

**Published:** 2013-08-09

**Authors:** Mirriam E. Nyenje, Ezekiel Green, Roland N. Ndip

**Affiliations:** 1 Department of Biochemistry and Microbiology, Faculty of Science and Agriculture, University of Fort Hare, PMB X1314, Alice 5700, South Africa; E-Mails: nyenjem@yahoo.com (M.E.N.); egreen@ufh.ac.za (E.G.); 2 Department of Microbiology and Parasitology, Faculty of Science, University of Buea, Box 63, Buea, Cameroon

**Keywords:** *Enterobacter cloacae*, biofilm, growth medium, coaggregation, autoaggregation, temperature

## Abstract

This study evaluated the effects of growth medium, temperature, and incubation time on biofilm formation by *Enterobacter cloacae* strains. The ability to adhere to a surface was demonstrated using a microtiter plate adherence assay whereas the role of cell surface properties in biofilm formation was assessed using the coaggregation and autoaggregation assays. The architecture of the biofilms was examined under scanning electron microscope (SEM). All the strains adhered to the well of the microtiter plate when incubated for 48 h, irrespective of the growth medium and incubation temperature. It was also noted that 90% and 73% of strains prepared from nutrient broth and cultured in brain heart infusion (BHI) broth and tryptic soy broth (TSB), respectively, were able to form biofilms, in contrast to 73% and 60% strains from nutrient agar and cultured in BHI and TSB respectively grown under similar conditions. However, no statistically significant difference was observed when the two methods were compared. The coaggregation index ranged from 12% to 74%, with the best coaggregate activity observed when partnered with *Streptococcus pyogenes* (54%–74%). The study indicates the suitability of BHI and TSB medium for the cultivation of *E. cloacae* biofilms, however, temperature and incubation time significantly affect biofilm formation by these bacteria.

## 1. Introduction

*E. cloacae*, a Gram negative bacterium, belongs to the family *Enterobacteriaceae*. The bacterium comprises part of the normal flora of the gastrointestinal tract of 40%–80% of the human population and is widely distributed in the environment [1]. The organism has also been isolated from food processing plants, fresh vegetables, rice, meat and meat products [2,3,4]. *E. cloacae* are capable of causing opportunistic infections including pneumonia, urinary tract, wound, skin and soft tissue, ophthalmic and bloodstream infections, particularly catheter-related where it has been reported to form biofilms in dwelling catheters of hospital patients [5,6].

Biofilms are defined as matrix-enclosed bacterial populations, which adhere to each other and to surfaces; observations have conclusively shown that biofilm bacteria predominate numerically and metabolically, in virtually all ecosystems and are difficult to eliminate [7]. Bacterial biofilms are known to be linked to medical conditions such as cystic fibrosis, periodontitis, and nosocomial infections from the use of catheters and prosthetic heart valves [8]. However, biofilm formation in food processing plants has also been documented; both pathogenic and food spoilage microorganisms have been isolated from such bacterial communities [9].

It is well known that bacteria, including foodborne pathogens grow predominantly as biofilms in most of their natural habitats, but also in food processing, catering and the domestic environment [10]. Biofilms may generate a persistent source of contamination, leading to serious hygienic problems and also economic losses due to food spoilage [11]. Poor sanitation of food-contact surfaces is believed to be an essential contributing factor of foodborne disease and development of bacterial biofilms. The formed biofilms are a source of cross contamination as cells will continuously detach and contaminate food once it passes over contaminated surfaces or through aerosols originating from contaminated equipment. This may seriously affect the quality and safety of the processed food and pose a potential risk to the consumer [12].

*E. cloacae* and other species of *Enterobacter* have been reported to cause foodborne illnesses through consumption of a variety of foods, including infant foods [2]. In our recent study, *E. cloacae* was reported to be the second most prevalent organism from ready-to-eat foods [4]; implying that the organism might be one of the emerging foodborne pathogens that can be endemic in food processing environments. Studies on the ability of *E. cloacae* to form biofilms have focused mostly on clinical isolates [13,14] where authors have hypothesized that their ability to persist in these environments as well as their virulence, is a result of their capacity to colonize medical devices [14]. However, in the food industry, studies have demonstrated the ability of *Cronobacter sakazakii* to adhere to feeding bottles, feeding tubes and other food processing surfaces with sparse information on *E. cloacae.* Modern food processing supports and selects for biofilm forming bacteria on food-contact surfaces due to mass production, lengthy production cycles and vast surface areas for biofilm development [15]. Biofilm formation depends on an interaction between three main components: the bacterial cells, the attachment surface and the surrounding medium [16]. However, environmental factors such as pH, temperature, osmolarity, O_2_ levels, nutrient composition and the presence of other bacteria also play important roles [17,18]. The integration of these influences ultimately determines the pattern and behavior of a given bacterium with respect to biofilm development [19]. Hence, biofilms are very diverse and unique, not just to the microorganism, but to the particular environment in which they are being formed. This makes *in vitro* characterization of biofilms difficult and requires the establishment of laboratory conditions that mimic the natural setting being studied. The ability of these organisms to form biofilms at ambient temperature (25 °C), which is mostly room temperature in tropical countries, implies that under favorable conditions, they may be able to adhere to kitchen utensils and other environments leading to cross-contamination of food processed in these areas. Therefore the present study aimed to determine the effects of temperature, nutrient content and incubation time on attachment and biofilm formation by *E. cloacae* strains at ambient (25 °C) and body (37 °C) temperature in BHI and TSB media; in an effort to ascertain the suitable biofilm growth conditions for this bacterium as a guide to its containment in the food industry. BHI was chosen as one of the nutrient-rich laboratory medium, and TSB a less-rich (compared to BHI) culture medium which are frequently used in biofilm investigation. 

## 2. Results and Discussion

### 2.1. Microtiter Adherence

Tests were performed in triplicates on three occasions, the results averaged and standard deviations were calculated. The cut-off was defined as three standard deviations above the mean OD of the negative control (ODc) which contained broth only. The following criteria were used to classify the different adherent strength: OD ≤ OD_C_ = non-adherent, OD_C_ < OD ≤ (2 × OD_C_) = weakly adherent; (2 × OD_C_) < OD ≤ (4 × OD_C_) = moderately adherent and (4 × OD_C_) < OD = strongly adherent [20].

*E. cloacae* isolates displayed four different biofilm phenotypes (non, weak, moderate and strong adherent) after incubation for 24 h in both BHI and TSB (Table 1). The optical density (OD) of non adherent (NA), weak adherent (WA), moderate adherent (MA) and strong adherent (SA) obtained when the bacteria were grown in BHI ranged from 0.359–0.486, 0.552–1.159, 1.092–2.14 and 2.192–4.22 respectively. On the other hand, the OD obtained when the bacteria were grown in TSB ranged from 0.352–0.618, 0.62–1.583, 1.25–2.414 and 2.542–3.938 for NA, WA, MA and SA respectively (Table 2). It was observed that all the strains (100%) adhered to the well of the microtiter plate when incubated for 48 h, irrespective of the growth medium (BHI or TSB) and incubation temperature (Table 1). On the other hand, adherence ability was demonstrated by 73% (22) and 60% (18) of the strains obtained from inoculums prepared in nutrient agar and grown in BHI and TSB respectively, compared to 90% (27) and 73% (22) prepared in nutrient broth grown in BHI and TSB respectively, when the plates were incubated at 25 °C for 24 h (Table 1). All the strains (100%) from nutrient agar cultures grown in BHI were able to form biofilm when incubated at 37 °C for 24 h compared to 28 (93%) from nutrient broth grown in BHI; no difference was noted from TSB cultures, as 93% of the strains from both nutrient agar and broth formed biofilm (Table 1). However, no statistical significance (*p-*value > 0.05) was observed when the two methods (agar and broth) were compared; thus it can be suggested that *E. cloacae* strains for biofilm formation assays may be cultivated either in broth or on solid medium.

Worthy of note is the fact that infecting bacteria are often surface-associated, and their cell surface can therefore be expected to be more similar to that of bacteria grown on solid medium than to that found grown in liquid media [13]. Bacterial cells grown on solid medium differ in expression of cell-associated molecules compared to those grown in liquid medium [20,21]. Probably the presence of slime layers that are regarded as soft polyelectrolyte layers surrounding the bacteria decreases the energy barrier due to electrostatic repulsion in the interaction of the organisms with negatively charged substrata and, thus, plays an important role in their adhesion [21]. In their study, Kiers *et al*. [21] found that cell surface softness of *Staphylococcus epidermidis* first grown on agar medium increased by a factor of two, compared to the cell surface softness for the strains grown in liquid medium. Therefore, preparation of the inoculum from broth or agar may directly influence the biofilm production, since adhesion is the first step in biofilm formation. 

It was also noted that a good number of strains grown in nutrient broth, 90% and 73% cultured in BHI and TSB respectively were able to form biofilm, in contrast to 73% and 60% strains prepared from agar grown under similar conditions. However, no statistical significance was observed (*p* > 0.005) when the two methods were compared.

Composition of the medium has been documented to influence the ability of bacteria to produce biofilm under *in vitro* conditions; in particular the presence of glucose in the growth medium (*i.e.*, standard TSB medium commonly contains 0.25% glucose) has been reported to enhance biofilm formation [22]. Some studies have shown preference for BHI to TSB, although some strains of *Staphylococcus*, *Vibrio* and *Pseudomonas* species have been reported to produce greater biofilm quantities in TSB while others do so in BHI [22,23]. The present study compared the two growth media and observed no statistical significance (*p* > 0.005). However, a slightly higher number of *E. cloacae* strains grown on BHI demonstrated the ability to adhere to the wells of the microtitre plate, implying that although both BHI and TSB media could support the development of *E. cloacae* biofilms *in vitro*, some strains will produce strong biofilms on BHI than TSB. These findings are in agreement with other studies [17,24], which found that supplementation (enriching) of TSB medium with glucose increased the ability of *Staphylococci* to form biofilms, while Knobloch *et al*. [25] recommended the use of the two media to confirm a biofilm positive isolate.

All (100%) of the *E. cloacae* strains demonstrated adherence characteristics at the low temperature of 25 °C when incubated for 48 h in comparison to the range of 60%–90% of the strains that had the same characteristics when incubated for 24 h only; the range increased with high temperatures (37 °C) where 93%–100% of the strains demonstrated the adherence ability when incubated for 24 h (Table 1). Statistical significant adherence was observed (*p* < 0.005) when the two incubation periods were compared, which may imply that long incubation times and high temperatures influence biofilm formation.

Previous works have indicated that temperature, nutrients and other components in media affect attachment of microorganisms to surfaces of various materials [26,27]. The present study observed strong biofilms from plates incubated at 37 °C (Table 1). This could be attributed to the fast growth rate of the bacteria at higher temperatures [23]. Similarly, Iversen *et al*. [27] reported strong adherence of their test bacteria at high temperatures (>40 °C). 

**Table 1:** The effects of temperature, incubation time and growth medium on the biofilm formation of *E. cloacae* isolates.

B. phenotype	Parameters number (%)															
	BHI ^a^				TSB ^a^				BHI ^b^				TSB ^b^			
	24 h		48 h		24 h		48 h		24 h		48 h		24 h		48 h	
	25 °C	37 °C	25 °C	37 °C	25 °C	37 °C	25 °C	37 °C	25 °C	37 °C	25 °C	37 °C	25 °C	37 °C	25 °C	37 °C
NA	8(27)	0	0	0	12(40)	2(7)	0	0	3(10)	2(7)	0	0	8(27)	2(7)	0	0
WA	18(60)	10(33)	10(33)	5(17)	14 (47)	17(57)	18(60)	4(13)	20(67)	11(37)	12(40)	4(13)	19(63)	14(47)	6(20)	2(7)
MA	4(13)	18(60)	16(53)	0	4 (13)	10(33)	11(37)	10(33)	7(23)	14(47)	15(50)	9 (30)	3 (10)	11(37)	16(53)	11(37)
SA	0	2(7)	4(13)	25(83)	0	1 (3)	1 (3)	16(53)	0	3(10)	3(10)	17(57)	0	3(10)	8(27)	17(57)
T. adh. (%)	22 (73)	30 (100)	30 (100)	30 (100)	18 (60)	28 (93)	30 (100)	30 (100)	27 (90)	28 (93)	30 (100)	30 (100)	22 (73)	28 (93)	30 (100)	30 (100)

BHI, brain heart infusion broth; TSB, tryptic soya broth; NA, non adherent; WA, weak adherent; MA, moderate adherent; SA, strong adherent; T. dh., total adherent; B. phenotype, biofilm phenotype; BHI ^a^, colonies from Nutrient agar and grown in BHI broth; TSB ^a^, colonies from Nutrient agar and grown in TS broth; BHI ^b^, inoculums from Nutrient broth and grown in BHI; TSB ^b^, inoculums from Nutrient broth and grown in TSB.

**Table 2:** The mean optical densities and standard deviations of the different biofilm phenotypes.

Parameters	Biofilm formation							
	Non-adherent		Weakly adherent		Moderately adherent		Strongly adherent	
	OD range	Mean OD ± SD	OD range	Mean OD ± SD	OD range	Mean OD ± SD	OD range	Mean OD ± SD
25 °C BHI (24 h) ^a^	0.448–0.486	0 0.470 ± 0.02	0.572–0.990	0.733 ± 0.13	1.30–2.14	1.406 ± 0.15	ND	ND
25 °C BHI (48 h) ^a^	ND	ND	0.611–1.06	0.875 ± 0.13	1.12–2.01	1.344 ± 0.25	2.192–2.769	2.543 ± 0.39
25 °C TSB (24 h) ^a^	0.352–0.618	0.512 ± 0.08	0.626–1.131	0.785 ± 0.15	1.365–1.458	1.405 ± 0.07	ND	ND
25 °C TSB (48 h) ^a^	ND	ND	0.625–1.237	0.984 ± 0.18	1.625–1.789	1.507 ± 0.231	ND	ND
37 °C BHI (24 h) ^a^	ND	ND	0.590–1.074	0.885 ± 0.12	1.164–2.022	1.504 ± 0.34	2.224–3.327	2.775 ± 0.77
37 °C BHI (48 h) ^a^	ND	ND	0.580–0.784	1.872 ± 0.32	1.331–2.13	1.872± 0.32	2.35–4.22	3.034 ± 0.62
37 °C TSB (24 h) ^a^	0.421–0.464	0.442 ± 0.03	0.662–1.081	0.861 ± 0.169	1.297–1.997	1.591 ± 0.199	ND	ND
37 °C TSB (48 h) ^a^	ND	ND	0.988–1.583	1.483 ± 0.493	1.93–2.485	2.149 ± 0.190	2.556–3.635	2.955 ± 0.503
25 °C BHI (24 h) ^b^	0.359–0.462	0.420 ± 0.05	0.565–1.083	0.832 ± 0.14	1.098–1.988	1.509 ± 0.340	ND	ND
25 °C BHI (48 h) ^b^	ND	ND	0.729–1.048	0.855 ± 0.13	1.436–2.144	1.728 ± 0.584	2.253–3.24	2.660 ± 1.13
37 °C BHI (24 h) ^b^	0.44–0.481	0.460 ± 0.02	0.552–1.111	0.803 ± 0.17	1.123–2.039	1.577 ± 0.36	2.405–3.27	2.837 ± 0.61
37 °C BHI (48 h) ^b^	ND	ND	1.01–1.159	1.074 ± 0.06	1.392–2.129	1.697 ± 0.24	2.247–3.805	2.93 ± 0.546
25 °C TSB (24 h) ^b^	0.421–0.559	0.508 ± 0.04	0.62–1.117	0.834 ± 0.16	1.319–1.774	1.548 ± 0.22	ND	ND
25 °C TSB (48 h) ^b^	ND	ND	0.711–1.066	1.034 ± 0.18	1.364–2.414	1.706 ± 0.36	2.568–3.382	2.929 ± 0.36
37 °C TSB (24 h) ^b^	0.503–0.535	0.519 ± 0.2	0.623–1.153	0.877 ± 0.2	1.561–2.413	1.802 ± 0.2	2.542–2.66	2.601 ± 0.08
37 °C TSB (48 h) ^b^	ND	ND	0.841–1.076	0.958 ± 1.66	1.25–2.19	1.831 ± 0.54	2.612–3.938	3.261 ± 0.54
OD, optical density; SD, standard deviation; ND, not determined; ^a^, colonies from Nutrient agar; ^b^, inoculums from Nutrient broth; BHI, brain heart infusion broth; TSB, tryptic soy broth.								

**Table 3:** Coaggregation indices of selected biofilm forming *E. cloacae* strains with seven different reference strains partners.

Isolate (biofilm phenotype)	%Autoagg.	Coaggregation indices (%)							
			Partner strains						
		Range %	*S. aureus* NCTC 6571	*S. pyogenes* A ATCC 49399	*S.* Typhimurium ATCC 13311	*P. aeruginosa* ATCC 15442	*P. shigelloides* ATCC 51903	*A. hydrophila* ATCC 35654	*S. sonnei* ATCC 29930
EC 52-2 (NA)	27	55–66	55	66	63	55	64	61	58
EC 205 (NA)	38	54–66	54	63	66	62	62	64	61
EC 12-2 (WA)	45	56–69	69	69	59	56	68	69	ND
EC 70-2 (MA)	89	12–70	57	61	29	70	12	64	56
EC 89-2 (MA)	30	31–62	43	54	62	40	53	31	23
EC 235 (WA)	34	65–74	68	74	74	72	72	65	69

NA, non adherent; WA, weak adherent; MA, moderate adherent; Autoagg, autoaggregation.

Contrary to these findings were the observations by Di Bonaventura *et al*. [18] who reported a higher biofilm production at a lower temperature (32 °C) than the higher temperatures of 37 °C; which may further suggest that biofilm formation varies with temperature and organism. Hence from the findings of the current study it can be deduced that *E. cloacae* strains will produce strong biofilms at higher temperatures.

### 2.2. The Capability of *E. cloacae* Strains to Autoaggregate and Coaggregate with Partner Organisms

All *E. cloacae* strains were able to coaggregate with the seven partner strains; the coaggregation index ranged from 12%–74% with best coaggregate activity observed when partnered with *Streptococcus pyogenes* (54%–74%) (Table 3). It was also noted that most of the strains which weakly adhered to the wells had a slightly higher coaggregation index range of 56%–74% followed by moderate adherent 12%–70% and the least was non- adherent with 54%–66% (Table 3). Of particular interest was a weakly adherent isolate, EC 235 which demonstrated the strongest coaggregation with all the partners. On the other hand, all the strains were able to adhere to each other, with autoaggregation range of 27%–89% (Table 3).

**Figure 1:**
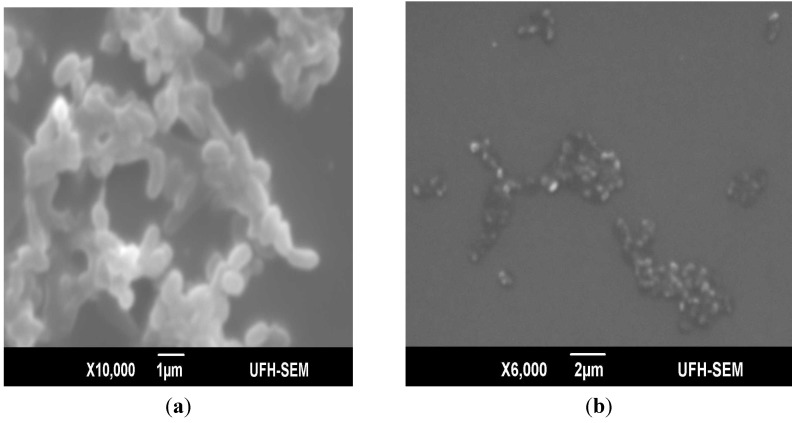
Scanning electron micrographs of an autoaggregate and coaggregate biofilm of *E. cloacae* and its coaggregate partner *P. aeruginosa* ATCC 15442 (**a**) strong adherent autoaggregate; (**b**) coaggregate of *E. cloacae* and partner *P. aeruginosa* ATCC 15442.

First recognized between bacterial isolates from human dental plague, coaggregation is the highly specific recognition and adhesion of different bacterial species to one another by specific molecules. Coaggregation in biofilms is important in development of mix-culture biofilms; it enables cells to withstand the highly changing environment, which on the other hand can adversely affect mono-dispersed cells. The results of this study revealed that *E. cloacae* isolates which weakly adhered to the microtiter plate demonstrated the strongest coaggregation with all the seven partner strain; these findings suggest the possibility of these strains to act as bridging organisms in multi-generic biofilms due to their ability to coaggregate with diverse coaggregating partners. Such bridging organisms are believed to carry complementary receptors recognized by functionally similar adhesins on cells from distinct genera [28]. Figure 1 shows the scanning electron micrographs of *E. cloacae* autoaggregates and its coaggregate partner *Pseudomonas aeruginosa* ATCC 15442. The SEM graphics of coaggregate assay demonstrated strong coaggregation with *P. aeruginosa.* It was also observed that *E. cloacae* strains coaggregated with organisms important in food spoilage and/or intoxications, *i.e.*, *S. aureus*, *S.* Typhimurium, *Shigella sonnei* and *P. aeruginosa* implying that *E. cloacae* in food processing plants can enhance the bridging of organisms in multi-generic biofilms.

## 3. Experimental

### 3.1. Bacterial Strains

Bacterial strains used in the study consist of *E. cloacae* previously isolated from various food sources [4] and reference strains*: S. pyogenes* A ATCC 49399, *Plesiomonas shigelloides* ATCC 51903, *P. aeruginosa* ATCC 15442, *S.* Typhimurium ATCC 13311, *Aeromonas hydrophila* ATCC 35654 and *S. sonnei* ATCC 29930. These strains have been reported to form biofilms [29,30,31,32]; and were also available in our laboratory at the time of the experiments.

### 3.2. Microtiter Plate Assay

*E. cloacae* isolates were screened for their adherence to polystyrene microtiter plate wells following 24 and 48 h incubation at 25 °C or 37 °C, in BHI and TSB. The method previously described by Nyenje *et al*. [33] was employed which made use of the agar and broth techniques; for the agar method, *E. cloacae* isolates were cultured on Nutrient agar (Oxoid, Basingstoke, England) and plates incubated at 37 °C for 24 h. Few single colonies were suspended in sterile saline to a turbidity standard comparable to a 0.5 McFarland. The suspension was vortexed for 1 min from which 20 µL was pipetted into a 96-well U-bottomed microtiter plate (Greiner Bio-one GmbH, Frickenhausen, Germany) containing 180 µL of BHI or TSB (Oxoid). With the inoculum for broth technique, isolates were grown in Nutrient broth (Oxoid), incubated for 24 h at 37 °C. Subsequently, the bacterial suspension was diluted 1:100 in BHI or TSB; an aliquot of 200 μL of diluted bacterial suspension was added to each well. The negative control wells contained 200 mL of broth only per well. The plates were incubated aerobically at different temperatures (25 °C and 37 °C) for 24 and 48 h. At the end of incubation period, the wells were carefully aspirated and washed three times with 200 µL of sterile normal saline to remove the unbound bacteria. The plates were left to dry overnight, fixed with hot air at 65 °C for 1 h, and the wells were stained with 150 µL of 1% crystal violet for 30 min. The plates were carefully rinsed off under running tap water to remove excess stain, left to dry at room temperature before resolubilizing the dye bound cells with 150 µL of 33% (v/v) glacial acetic acid. The optical density (OD) of each well was measured at 595 nm using a microtiter plate reader (SynergyMx, Biotek^TM^ Winooski, VT, USA). Reference strains of *P. aeruginosa* ATCC 15442 and *S. aureus* NCTC 6571 were used as positive controls because they have been widely reported to form biofilms in various environments [29,30,31,32].

### 3.3. Autoaggregation and Coaggregation Assays

A representative of the different biofilm phenotypes were examined for their ability to coaggregate with the following reference strains: *S. aureus* NCTC 6571, *S. pyogenes* A ATCC 49399, *S*. Typhimurium ATCC 13311, *P. aeruginosa* ATCC 15442, *P. shigelloides* ATCC 51903, *A. hydrophila* ATCC 35654, *S. sonnei* ATCC 29930. The individual strain was grown in 20 mL of BHI broth at 37 °C, harvested after 48 h and washed twice in 3mM NaCl containing 0.5 mM CaCl_2_. Subsequently, the cells were re-suspended in the same solution (3 mM NaCl containing 0.5 mM CaCl_2_), centrifuged at 650 × g for 2 min and the supernatant discarded; the OD of the cell suspension used for coaggregation assay was adjusted to 0.3 at a wavelength of 660 nm, using an automated spectrophotometer (Optima Scientific V-1200, Cambridge, UK). Equal volumes of the cell suspension (1 mL each) of *E. cloacae* and the coaggregating partners were mixed and the OD (OD_Tot_) immediately read at 660 nm before incubation at room temperature for 2 h. Subsequently, the tubes were centrifuged at 2,000 rpm for 2 min and the OD of the supernatant (OD_s_) measured at the same wavelength (660 nm) [20]. The degree of coaggregation of the paired isolates was determined using the equation:

% coaggregation = (OD_Tot_ − OD_s_) × 100/OD_Tot_

For autoaggregation assay, the individual bacterial suspension adjusted to an OD of 0.3 was incubated at room temperature for 1 h, centrifuged at 2,000 rpm for 2 min and the OD of the supernatant was measured at 660 nm. The degree of autoaggregation was calculated as follows:

% autoaggregation = (OD_0_ − OD_60_) × 100/OD_0_

OD_0_ refers to the initial OD of the organism, and OD_60_ is the OD of the supernatant after 60 min of incubation.

### 3.4. Characterization of Biofilm Formation Using Scanning Electron Microscope

To evaluate the architecture of the formed biofilms, SEM was used in accordance with the method previously described by Nyenje *et al*. [33]. Briefly, the isolates were grown on a microscope cover slip (22 × 22 mm) in a Petri-dish half-filled with BHI broth and incubated at 37 °C for 72 h. For the coaggregation assay, the partner isolate was added after 1 h of incubation. At the end of the incubation, the cover slips were washed three times with normal saline before fixing with 2.5% (w/v) glutaraldehyde solution for 1 h. Subsequently, the samples were dehydrated in a series of 20, 40, 60, 80 and 99.5% ethanol solution for 30 min in each concentration before post fixing in 1% osmium tetroxide (OsO_4_). The samples were critical point-dried using CO_2_ and sputter-coated with gold palladium using an Elko 1B.3 ion coater before viewing with the SEM (Japan Electron Optical Laboratories JSM-6390LV, Tokyo, Japan).

## 4. Statistical Analysis

Statistical analysis was performed using SPSS version 19. One way ANOVA followed by Turkey’s *post hoc* test was used to compare the agar and broth methods, BHI and TSB liquid media. The incubation temperatures and periods were also compared; the mean difference was considered significant at *p* < 0.05.

## 5. Conclusions

This study demonstrated that *E. cloacae* readily form biofilms on plastic surfaces, which are nowadays frequently used in food-processing environments, raising a great concern for the food industry as organisms in biofilms are difficult to eliminate, hence creating a reservoir for cross contamination. The study also indicated the suitability of BHI and TSB medium for the cultivation of *E. cloacae* biofilm however, temperature and incubation time significantly affected biofilm formation by these bacteria. 
